# Brazilian version of the body dysmorphic disorder examination

**DOI:** 10.1590/S1516-31802008000200005

**Published:** 2008-03-06

**Authors:** Renata Trajano Borges Jorge, Miguel Sabino, Jamil Natour, Daniela Francescato Veiga, Anamaria Jones, Lydia Masako Ferreira

**Keywords:** Body image, Somatoform disorders, Evaluation, Plastic surgery, Questionnaires, Imagem corporal, Transtornos somatoformes, Avaliação, Cirurgia plástica, Questionários

## Abstract

**CONTEXT AND OBJECTIVE:**

Body image improvement is considered to be the main reason for undergoing plastic surgery. The objective was to translate the body dysmorphic disorder examination (BDDE) into Brazilian Portuguese and to adapt and validate this questionnaire for use in Brazil.

**DESIGN AND SETTING:**

Cross-sectional survey, at the Department of Plastic Surgery of Universidade Federal de São Paulo.

**METHODS:**

The BDDE was first translated into Portuguese and then back-translated into English. These translations were then discussed by healthcare professionals in order to establish the final Brazilian version. In a second stage, the validity and reliability of the BDDE were assessed. For this, patients were initially interviewed by two interviewers and subsequently, by only one of these interviewers. On the first occasion, in addition to the BDDE, the body shape questionnaire (BSQ) and the Rosenberg self-esteem scale were also applied. These questionnaires were applied to 90 patients.

**RESULTS:**

Six questions were modified during the assessment of cultural equivalence. Cronbach's alpha was 0.89 and the intraclass correlation coefficients for interobserver and test-retest reliability were 0.91 and 0.87, respectively. Pearson's coefficient showed no correlation between the BDDE and the Rosenberg self-esteem scale (0.22), whereas there was a moderate correlation between the BDDE and the BSQ (0.64).

**CONCLUSIONS:**

The BDDE was successfully translated and adapted, with good internal consistency, reliability and construct validity.

## RESUMO

**CONTEXTO E OBJETIVO:**

A busca pela melhoria da imagem corporal é a principal motivação dos pacientes que procuram um cirurgião plástico. O objetivo deste estudo foi traduzir para o português, adaptar à cultura brasileira, testar a validade de construção e a reprodutibilidade do Body Dysmorphic Disorder Examination (BDDE).

**TIPO DE ESTUDO E LOCAL:**

Estudo transversal, realizado no Departamento de Cirurgia Plástica da Universidade Federal de São Paulo.

**MÉTODOS:**

O estudo foi realizado em duas fases. Primeiramente, o instrumento foi traduzido e retro-traduzido. Essas traduções foram submetidas à revisão por um comitê multidisciplinar até que se construísse uma versão consensual em Português. Na segunda fase do estudo foram testadas a validade e reprodutibilidade do instrumento. Os pacientes foram entrevistados em duas ocasiões distintas. Na primeira, por dois entrevistadores e na segunda ocasião por apenas um deles. Na primeira ocasião também foram administrados o *Body Shape Questionnaire* (BSQ) e a Escala de Auto-Estima de Rosenberg.

**RESULTADOS:**

Durante a fase de adaptação, seis questões foram modificadas. A consistência interna do instrumento foi de 0,89. O coeficiente de reprodutibilidade inter-observador foi de 0,91 e o intra-observador foi de 0,87. Quanto à validade, pode-se afirmar que os questionários BDDE e Rosenberg não apresentam correlação (0,22), ao passo que entre BDDE e BSQ a associação existente é moderada (0,64).

**CONCLUSÕES:**

O BDDE foi traduzido e adaptado com sucesso, demonstrando ser válido e reprodutível.

## INTRODUCTION

The development and validation of health-related quality-of-life questionnaires has become an important area of medical research, mainly because of the need to monitor and evaluate the treatment given to patients rather than simply dealing with the patient's complaint or illness. In this way, various instruments that allow objective assessment of individuals’ wellbeing have been created and translated into different languages.^1,2^

Body image improvement is considered to be the main reason why most patients undergoing plastic surgery do so.^3^ Traditionally, plastic surgery evaluation has generally been assessed using photographic documentation to compare the patient's appearance before and after surgery. However, there have been efforts to humanize this evaluation and make it less technical.^3–5^

The body dysmorphic disorder examination (BDDE) could provide an additional parameter for evaluating the success of plastic surgery and for assessing possible complaints by such patients. The BDDE is a specific quality-of-life instrument that deals solely with the patient's body image. The questionnaire includes 34 questions that evaluate the degree of dissatisfaction relating to a given physical feature and facilitate the diagnosis of body dysmorphic disorder. The BDDE is valid and reliable and has already been used in several languages.^6^

## OBJECTIVE

The aim of this study was to translate the BDDE into Brazilian Portuguese and to assess to its cultural equivalence, validity and reliability among Brazilian patients.

## METHODS

The original BDDE was translated from English to Brazilian Portuguese by two independent translators, as recommended by Guillemin et al.^7,8^ and Beaton et al.^9^ These two translations were analyzed by healthcare professionals (one rheumatologist, one physiotherapist and three plastic surgeons) in order to establish a consensual Brazilian Portuguese translation. This was then back-translated into English by two other translators, both fluent in English, who were unaware of the existence of the original questionnaire. These two English versions were then compared each other by the same health care workers in order to detect possible errors and to establish a consensual English back-translation. This was found to be grammatically and semantically equivalent to the original English version, thus allowing the Portuguese translation to be accepted as the final Brazilian Portuguese translation.

### Cultural equivalence

The final Portuguese version of the BDDE was applied by an interviewer to 30 patients aged 18 years or over who were selected consecutively at the plastic surgery outpatient clinic of Universidade Federal de São Paulo (Unifesp). The study protocol was approved by the Ethics Committee and all patients signed an informed consent statement.

After reading each question in the Portuguese version of the BDDE, the patients had to indicate whether they had understood the question and, if they had, they needed to explain in their own words what was being asked and suggest any modifications that could improve the clarity of the question. If more than 20% of the questions were found to be incomprehensible to the patients, they would be analyzed and modified by the same healthcare group and then applied to another 30 patients from the same clinic, until the patients clearly understood more than 80% of the questions. In all, 60 patients were interviewed in two phases for the purposes of assessing the cultural equivalence. This modified version of the BDDE was then submitted to a final evaluation by these healthcare professionals. Table 1 summarizes the clinical and demographic data for the patients studied.

**Table 1.** Clinical and demographic characteristics of the patients studied to assess cultural equivalence (phase I) and reliability (phase II) of the body dysmorphic disorder examination (BDDE)

	Phase I	Phase II
Age (years)	42.6	38.1
Gender (females: males)	52:8	30:0
Educational level (years)	9.85	11.53
Percentage with breast defect correction	40.0%	56.7%
Time required to apply the BDDE (min)	10.40	10.00

All data are reported as means.

### Validity and reliability

For this step, a new group of 33 preoperative patients from the same plastic surgery outpatient clinic, who had not participated in the cultural equivalence phase, was selected. Patients who missed one of the interviews or who underwent surgery before the second interview were excluded.

The patients were evaluated by means of three interviews. On the first occasion, the patients were interviewed twice, once by each of two interviewers, to assess interobserver reliability. On the second occasion, 7-15 days after the first two interviews, the patients were again interviewed by the first interviewer to assess intraobserver reliability. This time interval was used because we assumed that the patients’ complaints would not change over this interval.

The construct validity was assessed on the first occasion by the first interviewer who, in addition to the BDDE, also applied the body shape questionnaire (BSQ) and the Rosenberg self-esteem scale. Both the BSQ and the Rosenberg scale had already been validated in Brazil. The BSQ evaluates concerns relating to body shape and assists in diagnosing eating disorders,^10^ whereas the Rosenberg scale evaluates the individual's self-esteem.^11^

### Statistical analyses

The intraclass correlation coefficient (ICC) was used to assess the intraobserver and interobserver reliabilities and Pearson's correlation was used to assess the construct validity. Cronbach's alpha was used to assess the internal consistency of the BDDE.

## RESULTS

### Cultural equivalence

Assessment of the cultural equivalence led to modification of six questions. The questions modified were question 5, in which the word *graduate* was replaced by *inform*, question 8 in which the expression *get reassurance* was replaced by the phrase *look for support*, questions 19 and 20 in which the expression *negative self-evaluation* was replaced by *critical,* question 28 in which the expression *control your posture* was replaced by *modify body movements* and question 31 in which the word *unclothed* was replaced by *without clothes.*

### Psychometric properties

The mean time required for the questionnaire to be administered was 10 minutes. Thirty-three female patients were interviewed. Three patients were excluded. Table 2 shows the scores obtained for the Brazilian Portuguese version of the BDDE in interviews 1 and 2, which were held on the same day by two interviewers (to obtain the interobserver reliability) and on a separate (third) occasion by the first interviewer (to obtain the intraobserver reliability).

**Table 2.** Scores obtained for the Brazilian Portuguese version of the body dysmorphic disorder examination

	Interview 1	Interview 2	Interview 3
Mean	80.8	74.9	78.6
Minimum	29.0	23.0	13.0
Maximum	154.0	149.0	154.0

### Internal consistency

Cronbach's alpha coefficient was 0.892 (range: 0.823-0.939), thus indicating that the questionnaire was internally consistent (Table 3).

**Table 3.** Intraobserver and interobserver reliability of the body dysmorphic disorder examination (BDDE) determined by the intraclass correlation coefficient, and Cronbach's alpha coefficient (Cα) determined by bootstrapping

	Intraobserver	Interobserver	Cα
Mean	0.871	0.910	0.892
Minimum	0.750	0.820	0.823
Maximum	0.936	0.956	0.939

### Intraobserver reliability

The ICC for interviews 1 and 3 was 0.871 (range: 0.750-0.936), thus indicating agreement between the scores from the two interviews (Table 3).

### Interobserver reliability

The ICC for interviews 1 and 2 was 0.91 (range: 0.820-0.956), thus indicating concordance between the scores for the same interviewer (Table 3).

### Construct validity

Pearson's correlation coefficient was used to study associations between the BDDE, Rosenberg self-esteem scale and BSQ. The BDDE and Rosenberg self-esteem scale showed no correlation with each other, whereas the BDDE and BSQ showed moderate correlation (Table 4).

**Table 4.** Pearson's correlation coefficients between the body dysmorphic disorder examination (BDDE) and the Rosenberg self-esteem scale and between the BDDE and the body shape questionnaire (BSQ)

	Coefficient	Confidence interval	
BDDE and Rosenberg	0.229	-0.143	0.544
BDDE and BSQ	0.641	0.365	0.813

## DISCUSSION

The patient's opinion regarding the aesthetic improvements achieved following plastic surgery is an important aspect of postsurgical assessment.^4,5^ This evaluation can be done using a variety of approaches, including the BDDE, which is a specific quality-of-life instrument that deals solely with the patient's body image.

In this study, we translated the BDDE into Brazilian Portuguese and validated its reliability and reproducibility among a series of patients at a plastic surgery outpatient clinic in the city of São Paulo, Brazil. This is the first study to apply the BDDE in Brazil.

The BDDE was chosen for the present study because of its internationally established validity and reliability, because it contains fewer questions than other similar questionnaires and because it has already been used in other languages.^6^ There was no need to create a new instrument since several good quality questionnaires are already widely used by the international scientific community, and it would be difficult to make meaningful comparisons with other studies done using different instruments in other cultural settings. In addition, we chose to translate, adapt and validate the interview version of the BDDE, in order to circumvent difficulties associated with the high levels of illiteracy and poor education of most patients who are attended at public health institutions in Brazil, and to increase the number of people among whom the instrument can be applied.^12^

The mean time required to apply the questionnaire was 10 minutes, which compared favorably with the time of around 10 minutes needed to administer the short form-36 (SF-36), a generic health-related quality-of-life measurement.^13^ In contrast, the Rosenberg self-esteem scale requires only an average of two minutes for it to be applied.^11^

To assess the cultural equivalence of the translated BDDE, 60 patients were interviewed on two occasions. These patients had had a mean of 9.8 years of schooling. In contrast, the 694 patients who participated in the creation and validation of the original BDDE had a mean education of 14.3 years.^6^ The number of patients in our investigation was comparable with the 40 patients who were interviewed in two phases during the process of translating and validating the Brazilian version of the SF-36.^13^ Similarly, during the process of translating, adapting and validating the Roland-Morris questionnaire, 30 patients were interviewed to assess the cultural equivalence; in that case, more than 80% understanding was achieved in the first series of interviews, which meant that there was no need for a second phase.^14^

While assessing the cultural equivalence, the wording of some questions required modification, but without altering the meaning of the questions. These modifications were necessary because, in many cases, the terms or phrases were unfamiliar to the population that was interviewed, or the language had to be simplified because of the high percentage of functional illiteracy among the patients, i.e. individuals who knew how to read and write but still had difficulty in understanding the meaning of spoken and written words.^12^

The validity and reliability of the BDDE were tested by interviewing 30 patients. This number of patients was similar to the 40 patients used to validate the disabilities of the arm, shoulder and hand (DASH) questionnaire for upper limb evaluation^15^ and the 32 patients used to validate the Rosenberg self-esteem scale.^11^

The maximum score for the BDDE is 168 points. The mean score obtained on the first occasion was 80.8 for the first interviewer and 74.9 for the second interviewer. On the second occasion, the mean score was 78.6 for the first interviewer, which gave a test-retest reliability of 0.87, compared with 0.94 in the original BDDE validation study.^6^ The interobserver reliability was 0.91, which was close to the 0.99 reported by Rosen and Reiter.^6^ These values confirm that our results were compatible with those of the original study.^6^

The internal consistency of the questions was 0.89, which indicated that, although the questions had undergone some modifications, they had not lost their original meaning and continued to measure the same concept, i.e. body image. Rosen and Reiter^6^ reported a value of 0.93 in their original study, Duarte et al.^16^ obtained an average coefficient of 0.80 and Ciconelli et al.^13^reported a coefficient of 0.3-0.5.

To assess the construct validity, we based our analysis on reports of psychological traits among plastic surgery patients,^17–20^ and on selected instruments that evaluated concepts similar to those examined here, like the Rosenberg self-esteem scale^11^ and the BSQ.^10^ The correlation between the BDDE and the Rosenberg self-esteem scale was 0.22 whereas Rosen and Reiter^6^ reported a value of 0.43. This difference may have occurred because body image is only one of the factors that influence self-esteem evaluations. In contrast, the correlation between the BDDE and the BSQ was 0.64, which compared favorably with the 0.69 reported by Rosen and Reiter.^6^ The moderate correlation between the BDDE and the BSQ does not necessarily mean that these instruments measure the same concept, but rather, that the answers to many of the questions in these two questionnaires tended to be similar to each other.

## CONCLUSION

In conclusion, the Brazilian version of the BDDE questionnaire was reliable and valid when applied to Brazilian patients. However, future studies should assess the sensitivity of the questionnaire for detecting changes in patients’ state of health.
